# Microplastics and non-natural cellulosic particles in Spanish bottled drinking water

**DOI:** 10.1038/s41598-024-62075-2

**Published:** 2024-05-15

**Authors:** Virginia Gálvez-Blanca, Carlos Edo, Miguel González-Pleiter, Francisca Fernández-Piñas, Francisco Leganés, Roberto Rosal

**Affiliations:** 1 Department of Chemical Engineering, Universidad de Alcalá, 28871 Alcalá de Henares, Madrid Spain; 2 Department of Biology, Faculty of Science, Universidad Autónoma de Madrid, 28049 Madrid, Spain; 3 Centro de Investigación en Biodiversidad y Cambio Global (CIBC-UAM), Universidad Autónoma de Madrid, C. Darwin 2, 28049 Madrid, Spain

**Keywords:** Environmental chemistry, Environmental chemistry

## Abstract

This investigation explored the presence of microplastics (MPs) and artificial cellulosic particles (ACPs) in commercial water marketed in single use 1.5 L poly(ethylene terephthalate) bottles. In this work we determined a mass concentration of 1.61 (1.10–2.88) µg/L and 1.04 (0.43–1.82) µg/L for MPs and ACPs respectively in five top-selling brands from the Spanish bottled water market. Most MPs consisted of white and transparent polyester and polyethylene particles, while most ACPs were cellulosic fibers likely originating from textiles. The median size of MPs and ACPs was 93 µm (interquartile range 76–130 µm) and 77 µm (interquartile range 60–96 µm), respectively. Particle mass size distributions were fitted to a logistic function, enabling comparisons with other studies. The estimated daily intake of MPs due to the consumption of bottled water falls within the 4–18 ng kg^−1^ day^−1^ range, meaning that exposure to plastics through bottled water probably represents a negligible risk to human health. However, it's worth noting that the concentration of plastic found was much higher than that recorded for tap water, which supports the argument in favour of municipal drinking water.

## Introduction

Plastic pollution has become a global concern. The industry puts into the market an amount of plastic estimated at 400 million tonnes (Mt) per year, a figure that does not include polymers used for textiles, adhesives, or coatings^1^. The limited circularity of the plastic market is evident from the available data. In the EU27 (plus Norway, Switzerland, and the United Kingdom), 7.7 Mt of post-consumer plastics were reintroduced in the market out of a total production of 58.7 Mt in 2022. Using material flow analysis, the leakage of mismanaged plastic waste into the environment was estimated at 3.4 Mt in the EU in 2016^2^. Concerning global data, the Organisation for Economic Cooperation and Development (OECD) estimated that 22 Mt of mismanaged plastic wastes leaked to the environment worldwide in 2019^3^. Moreover, the forecasts indicate that the quantity of plastic entering ecosystems, especially aquatic ones, could potentially double within the coming years^4^.

Plastic waste entering the environment undergoes mechanical, oxidative, and photochemical degradation processes, resulting in progressively smaller fragments capable of dispersing and interacting with various biota, particles, and substances^5^. In a nomenclature inherited from marine studies, particles smaller than 5 mm in their larger dimension are referred to as microplastics (MPs) irrespective of their shape (fibres, fragments, or films) or origin (primary, already produced in that size or secondary, originated in the fragmentation of larger particles). Below 1 μm, particles resulting from the degradation of plastic objects that exhibit a colloidal behaviour are generally called nanoplastics (NPs)^6^. Eventually, MPs and NPs make their way to foods and beverages, exposing humans to a novel form of pollution whose health implications remain uncertain^7^.

Regarding drinking water, the presence of microplastics has become a growing concern for scientists, policymakers, and the general public due to its potential impact on human health^8^. MPs enter drinking water supplies, either from wastewater discharges or as a result of atmospheric deposition^9^. Once inside drinking water treatment plants, some MPs are so small that escape treatment processes such as sand and granular activated carbon filtration, ultimately making their way to final product^10^. In this regard, bottled water shares concerns with municipal drinking water sources. This is partly due to their shared origins; in certain countries, water from rivers is bottled after treatments like membrane filtration or deionization^11^. Additionally, the industrial processing required to produce bottled water provides opportunities for anthropogenic contaminants to enter the product. Specifically, the plastic materials used for bottles and food packaging containers raise concerns because of the possible leaching of plastic fragments and additives, especially in case of reuse of plastic drinking bottles^12^.

The concentration of MPs in tap water remains controversial, as studies report concentrations ranging from less than one particle per cubic metre to hundreds per litre, depending on the sampled particle size and water source^13–15^. A popular alternative to tap water is bottled water, but most studies indicate higher concentrations of MPs compared to tap water, especially when marketed in plastic bottles, which can be a solid argument in favour of municipal tap water wherever it constitutes an option^16^. However, the available results concerning MPs in bottled water also display significant variability, which complicates the intercomparison of studies. This variability primarily stems from the use of different methodologies, particularly the differences in size cut-off. However, even among studies employing similar protocols, including spectroscopic characterization and rigorous quality assurance/control procedures, these variations persist.

The potential harmful effects of plastics on human health are currently a topic of debate. The mechanisms through which plastic causes cellular harm include membrane damage, oxidative stress, inflammation, genotoxicity, and disruption of cellular processes. However, our understanding of the long-term toxicity of plastic particles and their associated chemicals, as well as their cumulative effects, remains limited. Furthermore, the cellular uptake, cytotoxicity, and potential harm caused by plastic particles are highly dependent on the cell type^17^. An additional challenge for conducting risk assessment stems from the difficulty in obtaining data on the actual exposure to MPs and NPs. Technical constraints in sampling and identifying small plastic particles hinder an accurate assessment of their concentration in the environment and the exposure of humans to them.

The aim of this investigation was to assess the significance of bottled water as a source of exposure to anthropogenic plastic pollution. Besides MPs, we evaluated the presence of artificial cellulosic particles (ACPs) in drinking water. ACPs represent an emerging class of anthropogenic pollutant, primarily consisting of cellulose fibres with evidence of industrial processing. Although ACPs pose comparable risks to MPs, particularly concerning their potential role in the transportation and fate of chemical pollutants, they have received significantly less attention^18^. In this study, we assessed for the first time the mass concentration of both pollutants, rather than solely determining their number concentration. This approach is key for deriving toxicologically relevant exposure data.

## Methods

### Sampling methodology

Bottled water (still, low mineralization) marketed in 1.5 L single use PET bottles from the five top-selling five brands, was purchased from local retailers by the consumers’ organization OCU (Organization of Consumers and Users of Spain). Still water in 1.5 L PET bottles is the most common format sold in the country and the five brands selected represented about 40% or the market share for that type of water in Spain. We sampled 45 L for each brand (225 L for all brands). In all cases, water was bottled in origin at their respective springs as indicated in their labels.

Water was stored in a dark place protected from extreme temperatures and filtered as soon as possible using Whatman Nuclepore Track-Etched Membranes, 25 mm, pore size 0.8 μm, made of black polycarbonate. Black filters were used to make it easy the visual identification of anthropogenic particles, many of which were white or transparent. We conducted vacuum filtration using multiple filters for each brand, filtering a set of bottles with each filter. Each bottle underwent filtration in three 0.5 L aliquots, with the cap being recapped after each aliquot. This process required the caps to be removed three times per bottle (and repositioned twice), replicating the typical handling of bottled water by consumers.

### Analyses

Immediately after filtration, all filters were stored in clean Petri dishes and dried at 60 °C for 24 h prior to visualization and analyses. Suspected particles of plastic or other anthropogenic pollutants were photographed and measured using a Euromex-Edublue stereomicroscope equipped with Image Focus software and kept in closed Petri dishes until chemical characterization. For it, particles from a random selection of potentially anthropogenic particles were individually picked up using metal tweezers or a needle, depending on their size, deposited on KBr discs, and spectroscopically analysed. Quality micrographs from selected particles were obtained using a Nikon Eclipse TI2-A microscope.

The identification of plastics and other artificial pollutants was carried out by means of micro-Fourier Transformed Infrared Spectroscopy (micro-FTIR) using a Nicolet iN5 FTIR Microscope coupled to a Nicolet iS20 FTIR Spectrometer (Thermo Fisher Scientific). The micro-FTIR equipment was operated in transmission mode in the 550–4000 cm^−1^ range with spectral resolution 8 cm^−1^. This procedure allowed obtaining high quality spectra for most particles, which were compared with the databases existing in software Omnic 9 (Thermo Scientific) and with our own databases, created with plastics from different origins by our group. Pearson correlation was used with a minimum of 70% matching for positive identification. Spectra with matching below 80% (but > 70%) were individually validated. From the total number of potentially anthropogenic particles, a random subsample was selected for spectroscopic characterization. The subset size was determined to achieve an accuracy threshold below 5% in polymer identification. Accuracy refers to the maximum difference between the true probability of the particle type and the subsampled probability, as explained elsewhere^19,20^. The concentration in the full sample was calculated by multiplying the number of positively identified particles by the ratio between the number of particles with the same typology and colour in sample and subsample.

In this work, we measured particles for which the smallest projected dimension was > 15 µm because this is the best spatial resolution that can be generally achieved when using mid-infrared light^21^. For particles smaller than 15 µm the restrictions imposed by the diffraction limit only allow obtaining good spectra in specific cases, which would result in a methodological bias^22^. Particles were classified as fibres, fragments, and films. Particles with aspect ratio equal to or greater than 3:1 (a boundary traditionally established for man-made mineral fibres) were considered fibres. If not, they were categorised as fragments except if one dimension was significantly lower than the other two, in which case they were classified as films.

### Mass and size distributions

In this work, the reference size for particles was the diameter of the sphere with the same volume as the particle, d_v_, which was computed from microscope images by means of the two main orthogonal projected dimensions, L and W, which corresponded to length and width for fragments and length and diameter for fibres. The representative size for fragments was calculated using the model proposed by Simon et al. that assumes ellipsoidal shape^23^. In this model the axes of the ellipsoid are L, W, and the third dimension perpendicular to the plane of the image, H, which is estimated assuming (H/W) = (W/L) taking (W/L) as the median value for all the fragments in the sample:

1 $$ d_{v} \; = \;\sqrt[3]{{L\,W^{2} \,\left( \frac{W}{L} \right)_{med} }} $$

Concerning films, d_v_ was estimated taking the third dimension as one-tenth of the smaller from the other two (W). The volume of fibres was calculated assuming cylindrical shape. Further information regarding the morphological characterization of particles based on two-dimensional images can be found elsewhere^24^. The mass of MPs and ACPs was calculated by summing up the masses of all individual particles, m_i_, using the average density for the corresponding polymers:

2 $$ m_{i} \; = \;\sum\limits_{i} {\frac{\pi }{6}} \,\rho_{i} \,d_{v,i}^{3} $$

The mathematical modelling of size distribution data involved fitting particle size, defined as d_v_, to a logistic function with the following cumulative frequency distribution:

3 $$ P\left( {d_{v} } \right)\; = \;\frac{1}{{1\; + \;\left( {\frac{{d_{v} }}{{d_{v,med} }}} \right)^{n} }} $$

where *d*_*v,med*_ was the median of size distribution. P(d_v_) is the probability of finding a particle with size higher than d_v_ in a given sample. The derivative of P(d_v_)*,* p(d_v_), is the probability density function that gives the probability of finding a particle within a given differential interval around d_v_. The probability density function allows for the estimation of mass (or mass concentration by dividing it by the sample volume) using an average density for polymer particles, as follows:

4 $$M_{{x_{1}  - x_{2} }} \; = \;N\;\int\limits_{{x_{1} }}^{{x_{2} }} {\frac{\pi }{6}\;\rho \;x^{3} \,p\left( x \right)\,d{\kern 1pt} x} \, = \;\;N\;\frac{\pi }{6}\;\rho \,\int\limits_{{x_{1} }}^{{x_{2} }} {x^{3} \,p\left( x \right)\,d{\kern 1pt} x}$$

where *N* is the total number (or number concentration) of particles in a sample. This equation will be used below to estimate the mass concentration of MPs from particle size distributions obtained from the literature. The integrals involved are easy to compute numerically and further details can be found elsewhere^15^.

### Estimated daily intake

The Estimated Daily Intake (EDI) due to the consumption of bottled water can be calculated using the following equation:

5 $$ EDI\; = \;\frac{C\; \times \;IR}{{BW}} $$

where *C* represents the concentration in µg/L or other mass concentration units, IR denotes the ingestion rate in L/day, and BW signifies the body weight of a given population segment. Statistical data indicates a global consumption of bottled water in Spain of 3.042 × 10^9^ L in 2021 (last year available), representing an average of 65 L per person per year (source: Spanish Ministry of Agriculture, Fisheries and Food). A body weight of 70 kg was assumed for adults, and 16 kg for children.

### Quality assurance and quality control

The measures taken during sampling and laboratory handling to ensure the quality of the data obtained followed the general recommendations stated by the World Health Organization and the prescriptions indicated in the AFNOR guideline XP T 90-968-1 Water quality—Analysis of microplastics in human drinking water and groundwater—Part 1: Methods using vibrational spectroscopy^22,25^. All materials used in this work were made of metal and glass and cleaned with Milli-Q water (equipped with Millipak 0.22 µm membrane filter), wrapped with aluminium foil, and heated to 450 °C for 4 h to remove all possible rests of organic matter or any contamination from plastics or other artificial fibres. All processing was performed in a laminar flow hood with HEPA filter carefully cleaned before any operations. Procedural controls were deployed as indicated below. Laboratory clothes were made of purple-dyed cotton to easily recognize that source of pollution.

During sampling and identification, clean filters were deployed in open Petri Dishes as procedural sampling control. In addition, 2 L of Milli-Q water were filtered twice through 0.8 µm filters to serve as control blanks for assessing possible contamination of water and laboratory devices. This process was repeated for every brand of bottled water. The total number of particles found in controls was 14 (1 fragment and 13 fibres), which are detailed in Supplementary Table S1, Supplementary Information, SI). All fibres were compatible with laboratory purple clothes and were not considered. Although transparent instead of black, one polycarbonate fibre was disregarded due to possible contamination with the material filters were made of. No other coincidence in colour and composition existed with the particles positively identified in this work as plastics or artificial non-plastic particles. The verification of the recovery rate of MPs was assessed using PET fragments in the size range of the sample (50% in the 75–150 µm) added to Milli-Q water and filtered as indicated below. The recovery rate obtained was > 94%.

## Results

The total number of potentially anthropogenic particles recovered was 1480. The random subsample subject to spectroscopic characterization consisted of 621 particles (423 fibres, 191 fragments, and 7 films). Among the 621 particles analysed, 81 were positively identified as plastics (synthetic polymers, matching ≥ 70%), 180 were classified as ACPs (all of them cellulose, matching ≥ 70%), 126 were considered possibly natural (all of them cellulose, mostly fibres with insufficient evidence of having undergone industrial processing, although most of them are probably textile fibres). Additionally, 6 particles corresponded to calcium carbonate. The composition of the remaining particles (228) could not be unambiguously assessed (matching < 70%).

**Figure 1:**
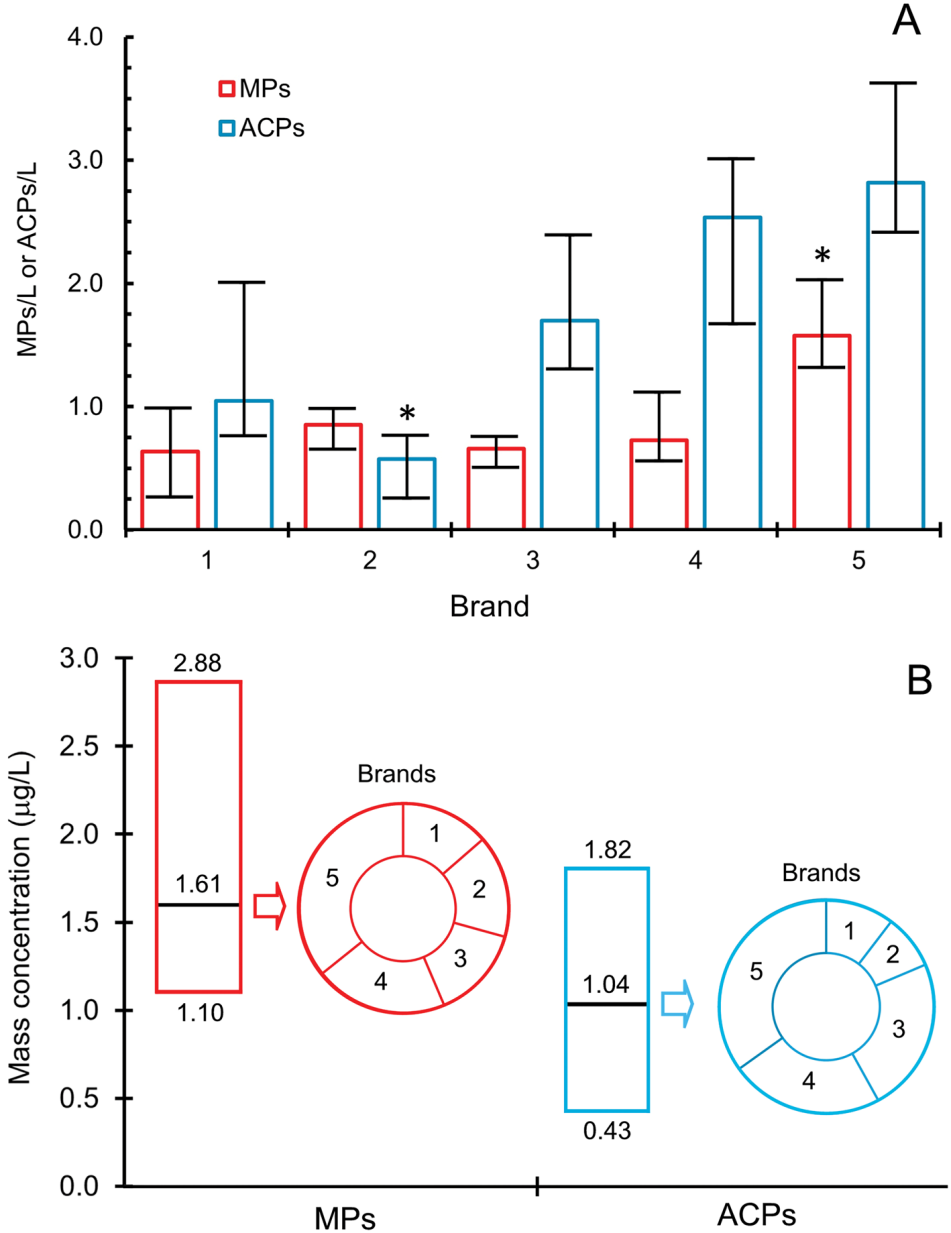
Number (**A**) and mass (**B**) concentrations of MPs (red) and ACPs (blue) in the five bottled water brands analysed in this study. The error bars in (**A**) indicate the range of values obtained from the filters used for each brand. The asterisks represent significant differences assessed by means of a Mann–Whitney U Test (*p*-value < 0.05). The boundaries of (**B**) represent the maximum and minimum concentrations observed among brands, which are represented as proportional sectors.

Figure 1A,B show, respectively, the number and mass concentration of MPs and ACPs in the five brands studied in this work. The median number concentrations were 0.73 (0.64–1.58) MPs/L and 1.70 (0.58–2.82) ACPs/L, respectively. The numbers in brackets represent the lower and higher values for individual brands. A Kruskal–Wallis test was used to assess statistically significant differences between brands (*p*-value < 0.05). Wherever significant, pairwise comparisons were performed using the Mann–Whitney U Test (*p*-value < 0.05). Significant differences were limited to Brand 5 (significantly higher number concentration of MPs with respect to the rest) and Brand 2 (significantly lower number concentration of ACPs with respect to the rest) and are indicated as asterisks in Fig. 1A. The bars represent the range of values obtained from the filters used for each brand. Concerning mass concentrations (Fig. 1B), the averages were 1.61 (1.10–2.88) µg/L for MPs and 1.04 (0.43–1.82) µg/L, for ACPs. No significant differences in mass concentration were observed among brands.

**Figure 2:**
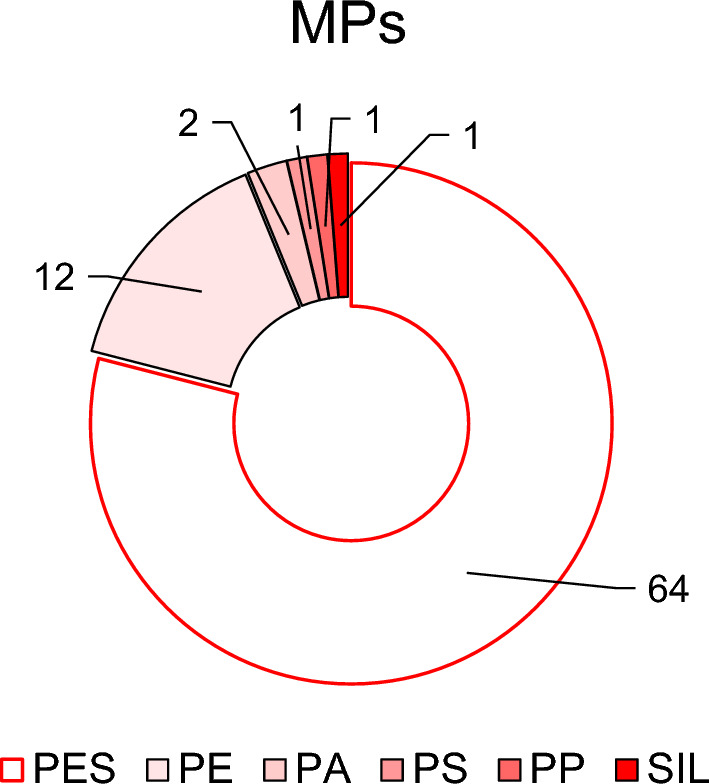
Chemical composition of the MPs identified in this work. (The numbers correspond to the ocurrence of the different types of plastic particles.)

The 81 MP particles identified comprised 29 fibres, 51 fragments and 1 film. Their composition, shown in Fig. 2, was largely dominated by polyester (PES, 35 fragtments and 29 fibres), polyethylene (PE, 11 fragments and 1 film), polyamide (PA, 2 fragments), polystyrene (PS, 1 fragment), polypropylene (PP, 1 fragment) and polysiloxane (SIL, 1 fragment).

**Figure 3:**
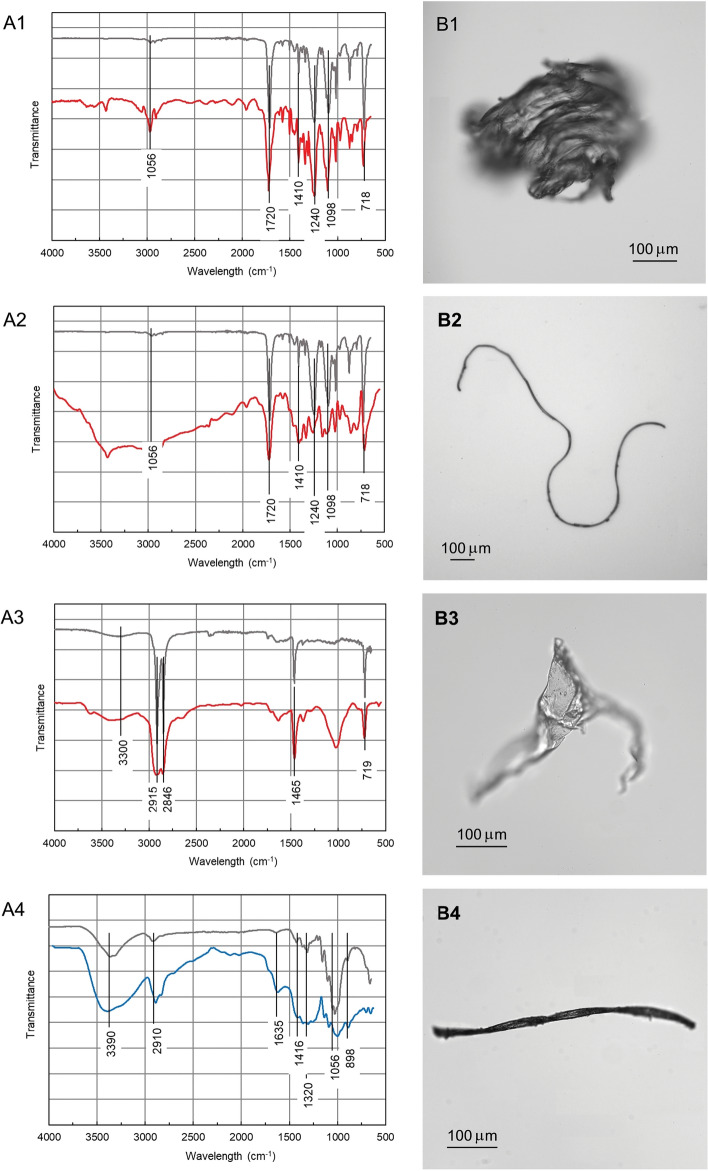
FTIR spectra (**A**) and micrographs (**B**) of a PES fragment (1), a PES fibre (2), a PE fragment (3) and a cellulose red fibre (4). Light grey: reference spectra. Scale bar: 100 µm.

Figure 3 shows micrographs of representative anthropogenic particles, namely a PES fragment, a PES fibre, a PE fragment, and a red cellulose fibre together with the infrared spectra that allowed their identification.

The colours of MPs and ACPs are depicted in Supplementary Fig. S1 (SI). While the majority of MPs were white or transparent, ACPs exhibited a variety of colors. All ACPs consisted of cellulose, predominantly in fibre form (with the exception of 4 fragments—2 red and 2 blue—and 2 films—1 red and 1 blue). White or transparent cellulose fibres were likely of textile origin, but they were not categorized as ACPs due to insufficient evidence confirming their artificial nature.

**Figure 4:**
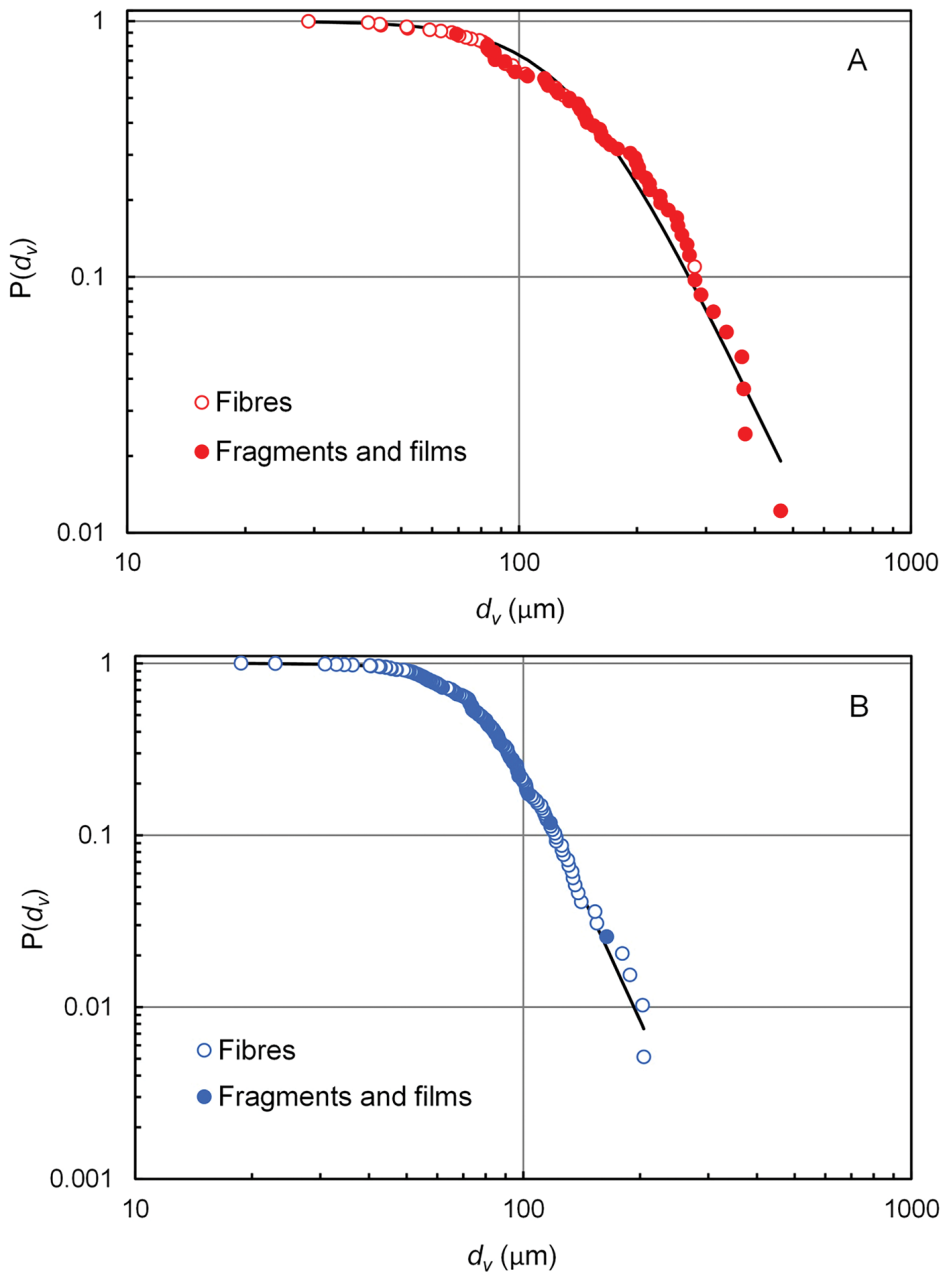
Size distribution of MPs and ACPs. Fibres are represented with empty circles and fragments and films with filled circles. The solid line corresponds to the fitting to a logistic function (Eq. 3). P(d_v_) is the probability of finding a particle with size higher than d_v_ in a given sample, dv being the diameter of the sphere with the same volume as the particle.

Figure 4 shows the cumulative size frequency distribution of MPs (A) and ACPs (B), which includes all the MPs and ACPs identified in this work. The solid lines represent the logistic fitting as defined in Eq. (3). The results for the fitting parameters are presented in Supplementary Table S2 (SI).

The median size of the particles found in this work was 93 µm for MPs (76–130 µm, interquartile range) and 76.7 µm for ACPs (60.4–96.0 µm, interquartile range). The smallest MP was a fibre with 12.5 µm diameter and 104 µm length (d_v_ = 29.0 µm); the smallest ACP was also a fibre with 10.0 µm diameter and 48.5 µm length (d_v_ = 18.7 µm). The largest MPs was a PE fragment (765 × 257 µm, d_v_ = 294 µm) and the largest ACPs a red cellulose fibre with 44 µm width and 2935 µm length (d_v_ = 204 µm).

Despite the higher number concentration of ACPs, the higher mass concentration for MPs was attributed to the size distribution of ACPs, which contained smaller particles, as depicted in Fig. 4A,B.

## Discussion

The available data regarding the presence of MPs in bottled water are quite scattered. Supplementary Table S3 (SI) presents literature findings that report sizes and concentrations from various types of bottles. Some studies use Raman spectroscopy because it offers the advantage of detecting particles with spatial resolution below the 10–20 µm limit typically found in micro-FTIR. The early report by Schymanski et al. used micro-Raman spectroscopy to identify MPs in returnable and single-use plastic bottles, as well as glass bottles and beverage cartons in containers sold in Germany. The authors found an average concentration of 118 ± 88 MPs/L in returnable bottles, which decreased to 14 ± 14 MPs/L in single-use plastic bottles^26^. In another early study also using micro-Raman, Oßmann et al. reported high concentrations of MPs in mineral water: 2649 ± 2857 MPs/L in single use PET bottles, and even higher, 8339 ± 7043 MPs/L, in reused bottles^27^. The difference could be attributed to the different size range of the particles identified. While in the first study, almost 80% of all microplastic particles found had a particle size between 5 and 20 µm, while Oßmann et al. detected smaller particles. However, the use of methodologies with different size span yield results difficult to compare. Specifically, it has been observed that micro-Raman studies tend to report higher particle abundances, which is an expected outcome in view of the well documented power law dependence of MPs abundance on particle size^28^. This methodological bias has also been observed in studies reporting MPs in water from drinking water treatment plants^15^. To address this issue, the approach followed in this work was to use probability distribution models using all size information available for every analysed particle as shown below.

Although most studies used spectroscopic characterization of microscopic particles, employing either micro-Raman or micro-FTIR techniques, some works limit their scope to identifying the larger particles accessible to ATR-FTIR. Others even omit any form of spectroscopic characterization. Mason et al. reported concentrations as high as > 10^4^ MPs/L; however, their identification method relied solely on Nile Red staining while mid-infrared spectroscopy was used only for particles ≥ 100 µm^29^. Lee et al. used a fluorophore other than Nile Red, specifically 1-pyrenebutyric acid *N*-hydroxysuccinimidyl ester. This compound was found to effectively stain various synthetic polymers and enabled the detection of MPs > 15 μm in commercial bottled water. The reported concentrations ranged from 6 to 58 MPs/L, with an overall size range of 45–723 µm^30^. The absence of spectroscopic (or thermo-analytical) information is an important shortcoming. Moreover, certain results offer room for methodological controversies due to questionable approaches. This is the case of using SEM to quantify MPs, which is a technique clearly inadequate to distinguish synthetic polymers from other particles^31,32^. The lack of standardized methods, the extrapolation from limited subsamples, and the lack of adequate quality assurance and quality control (QA/QC) information are common issues encountered in certain studies. A comprehensive list with detailed explanations is available elsewhere^26^.

In this study, we also assessed the concentration of ACPs as an emerging class of anthropogenic pollutants. All ACPs detected consisted of cellulose, primarily in fibre form, and were classified as anthropogenic based on their non-natural colours or textures. Being industrially processed materials, ACPs can incorporate a wide range of additives, such as dyes, softeners, flame retardants, biocides, antistatic agents, and others^33^. These additives can be either natural or, more commonly, synthetic chemicals that have the potential to leach out from the fibres. Other studies also investigated the presence of natural fibres in bottled water. Zhou et al. identified numerous cellulose and polyamide fibres in Chinese bottled water brands, cellulose being twice as abundant as synthetic polymers like PET and PE and attributed their presence to contamination from synthetic clothes during bottle washing^34^. In another study, Li et al. used laser direct infrared (LDIR) to detect particle pollutants in bottled water sold in China and found that over two-thirds of them (49 particles/L) were cellulosic^35^. LDIR is a technique that enables automatic particle localization and allows the analysis of smaller particles compared to the transmission or reflectance modes of micro-FTIR equipment. The presence of cellulose as anthropogenic pollutant has been reported by Aleksander-Kwaterczak et al. who studied the content of particles, including microplastics, in commercial Polish bottled water. They found 87–188 particles per litre, 50% < 20 µm, 38% fibres, of which about 30% were compatible with cellulose^36^. In another recent paper, Socas-Hernández et al. found a concentration of up to 42.4 anthropogenic particles per litre of bottled water, mostly (79%) fibres predominating cellulose (86%) over synthetic polymers, even in PET bottles^37^. In our work, we obtained concentrations of ACPs in the 0.58–2.82 cellulosic fibres/L or 0.43–1.82 µg/L range. Besides, there was an average of 1.21 fibres/L that were not classified as artificial due to limited evidence. In summary, our study revealed a relatively high concentration of ACPs in bottled water highlighting that the presence of anthropogenic pollutants in bottled water is not solely attributable to the materials used in bottles and caps. It is also a consequence of the pervasive pollution resulting from the widespread use of plastics and other materials, such as industrial textiles.

The composition of plastics agreed with the findings of other studies, predominantly reporting polyester and polyolefins. This outcome was reasonably expected, given that plastic bottles were made from PET (a polyester), while caps were all of them made of PE. In our results, all PES fragments except one were white or transparent, and therefore compatible with PET from bottles. Polyolefins were also found to be common in glass bottles, possibly due to cap abrasion^27^. Winkler et al. explored the release of MPs from plastic bottles and caps due to mechanical stress during bottle squeezing and repeated openings and closings. Their study demonstrated the generation of a substantial number of MP particles (PET from bottle necks and HDPE from caps) after repeated bottle usage^38^. In another study, both solar ultraviolet radiation and mechanical abrasion were observed to expedite the degradation of plastic surfaces, leading to the release of plastic particles^39^. In our work we took three samples per bottle by opening them three times (and recapping twice) to simulate a normal usage. The bottles from the brands we sampled had caps of different colours and 5 out of 11 PE fragments were compatible with them. One brand had a plastic label, but no films of that type were observed in water samples. Overall, considering composition and colour, most of the MPs found were compatible with the materials the bottles and caps are made of. However, we still found a significant number of fragments and fibres that can only be explained as the result of a diffuse pollution from other sources, especially from textiles.

Obtaining mass concentrations is not a trivial issue and can lead to severe errors if the measurement does not take into account the size and shape of all particles^40^. The application of Eq. (4) allowed calculating the concentration of MPs or ACPs for any size range. Based on the data collected in this work, the mass concentration of MPs with size < 100 µm was 169 ng/L, whereas that of ACPs amounted to 426 ng/L. The mass concentration of ACPs < 100 µm was higher than that of MPs due to the smaller size of ACPs. The same calculation can be done using size distributions from the literature even in the absence of any mass concentration data. The results can be found in Supplementary Table S2 (SI). Using the data provided by Oßmann et al. for single use and reusable PET bottles the concentrations are 23 ng/L and 171 ng/L respectively, for the 0.4–10 µm range^27^. The same calculation using the distribution data from Schymanski et al. yielded 260 ng/L and 650 ng/L, respectively for single use and returnable bottles within the 5–100 µm size range^41^. Accordingly, the mass concentration of small MPs in new bottles converges to rather similar values for small MP particles. Interestingly, this concentration is higher than those reported for most micropollutants in drinking water resources. For example, Tröger et al. examined various substances including pesticides, pharmaceuticals, personal care products, food additives, and perfluoroalkyl substances. Their analysis revealing individual concentrations spanning from sub-ng/L levels to tens of ng/L, and a total concentration in treated drinking water around 50 ng/L^42^.

A relevant result from our study is that bottled water contains higher contamination due to MPs and ACPs compared to municipal water supplies. In a recent study, we determined the occurrence of MPs in municipal drinking water from different cities and found an average number and mass concentrations of 12.5 MPs/m^3^ and 45.5 ng/L, respectively, which represent two-to-tree orders of magnitude lower than those reported here for water marketed in plastic (PET) bottles. It is important to note that both sets of data, municipal and bottles water, are comparable because the analyses were performed in the same laboratory, with essentially the same methodology and with the detected particles spanning over similar size range^15^. It is important to remark the advantages of tap water over bottled water. It is not only cost-effective and environmentally friendly, reducing plastic waste and carbon footprint, but also rigorously monitored by health authorities, ensuring its safety. Our findings highlight that tap water exhibits considerably lower concentrations of MPs compared to bottled water.

The estimated exposure to microplastics through bottled water was assessed using EDI values calculated using in Eq. (5). Assuming body weight of 70 kg for adults, and 16 kg for children, the EDIs would amount to 4 and 18 ng kg^−1^ day^−1^, for adults and children respectively (these values would increase by one order of magnitude for individuals ingesting 2 L of bottled water daily, an amount usually recommended by health experts). These figures are much lower than those estimated by Zucarello et al., which were 40.1 and 87.8 µg kg^−1^ day^−1^ for adults and children, respectively. However, the mass concentration reported by the authors was unusually high (100–3000 µg/L), and EDI would decrease to 18 and 79 ng kg^−1^ day^−1^ using the median values for number concentration and MPs size provided by the authors^32^. Similar EDI values can be obtained using others’ data as evidenced from the results shown in Supplementary Table S2 (SI), which reasonably align with the EDIs reported for other micropollutants in drinking water^43^.

The relative importance of anthropogenic pollution can also be contextualized by comparing the levels of MPs and ACPs found in bottled water with those that could be expected from fibre and plastic pollutants in indoor environments as suggested elsewhere^44^. For it, we estimated the contamination that could receive a glass of water (8.5 cm diameter, 250 mL) exposed to atmospheric fallout for 20 min in an indoor environment. Deposition rates in homes have been reported in different studies. Soltani et al. measured deposition rates in the 22–6169 fibres m^−2^ day^−1^ range in Australian indoor house dust, from which 39% were MPs and the rest natural fibres or processed cellulose^45^. Jener et al. reported 0–5412 MPs m^−2^ day^−1^ (range) mostly fibres in households sampled during a 6 month period in the United Kingdom^46^. The aforementioned figures suggest a range of 0.75–1.71 MPs/L, a value similar to those reported here for MPs and ACPs in bottled water.

According to our data, it is very unlikely that the ingested plastic due to bottled water could exhibit adverse effects on humans, as it represents a concentration several orders of magnitude lower than that reported to induce damage in in vitro biological models^17,47^. Nevertheless, in vitro biological models possess important limitations. The main one is that they are generally unsuitable for chronic exposure studies whereas, in our daily life, we are constantly exposed to plastics over extended periods of time^48^. Besides, the available toxicological studies have been conducted using primary plastics of a single type, while we may find secondary particles of various types in bottled water. In this context, it is important to note that only plastic particles with very small sizes, in the few microns range or below, can be absorbed through the gastrointestinal tract^49^. These very small particles, mainly within the nanosize range (< 1 μm), are very challenging to monitor, as they fall outside the capabilities of the usual spectroscopic characterization methods. However, there is a general agreement that they should be produced by the weathering and fragmentation of larger plastics. Therefore, even if apparently harmless, the precautionary principle forces to be cautious with the risk posed by plastic pollutants in drinking water. While existing data on plastic concentration in drinking water may align, precise mass concentration estimations are essential for a deeper comprehension of the sources, distribution, and potential health impacts of MPs and related pollutants, including additives used in their manufacturing. It is imperative that governments and regulatory bodies support comprehensive studies to inform evidence-based policies regarding the delicate issue of pollutants in food products.

### Supplementary Information

Supplementary Information.
