# Supplementary material for: Microplastics and non-natural cellulosic particles in Spanish bottled drinking water
Source: Sci Rep. 2024 May 15;14:11089. doi: 10.1038/s41598-024-62075-2 (PMC11096351; doi:10.1038/s41598-024-62075-2)
Supplement: Supplementary file 1 — Supplementary Information. [file 41598_2024_62075_MOESM1_ESM.docx]

**Supplementary Information**

Microplastics and non-natural cellulosic particles in Spanish bottled drinking water

Virginia Gálvez-Blanca^1^, Carlos Edo^1^, Miguel González-Pleiter^2^, Francisca Fernández-Piñas^2,3^, Francisco Leganés^2,3^, Roberto Rosal^1,*^

^1^ Department of Chemical Engineering, Universidad de Alcalá, E-28871 Alcalá de Henares, Madrid, Spain

^2^ Department of Biology, Faculty of Science, Universidad Autónoma de Madrid, E-28049, Madrid, Spain

^3^ Centro de Investigación en Biodiversidad y Cambio Global (CIBC-UAM), Universidad Autónoma de Madrid. C Darwin 2, 28049 Madrid, Spain

* Corresponding author: roberto.rosal@uah.es

**Contents:**

**Table S1**. Particles found in controls.

**Figure S1**. Colours of the MPs and ACPs identified in this work.

**Table S2**. Fitting results of size distribution data fitted to logistic functions (Eq. 3, fitting plot shown in the figure below) and mass concentrations calculated from size distribution.

**Table S3**. Literature findings on MPs in detected in bottled drinking water. Error in standard deviation (SD) when disclosed, or 95% CI.

**Table S1**. Particles found in controls.

| **Brand** | **Typology** | **micro-FTIR** | **Action taken in affected samples** |
| --- | --- | --- | --- |
| [1] | 3 Pink/Purple fibres | Cellulose | These fibres were not considered due to possible origin in laboratory clothes |
| [3] | 4 Pink/Purple fibres | Cellulose | These fibres were not considered due to possible origin in laboratory clothes |
|  | White fragment | Polycarbonate | This fragment was not considered because filters were made of polycarbonate |
| [4] | 2 Pink/Purple fibres | Cellulose | These fibres were not considered due to possible origin in laboratory clothes |
| [5] | 4 Pink/Purple fibres | Cellulose | These fibres were not considered due to possible origin in laboratory clothes |


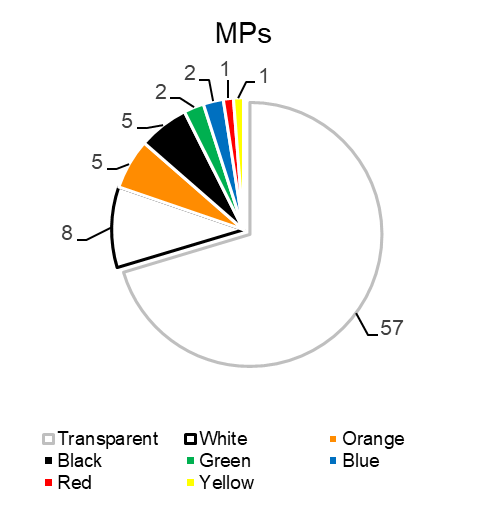

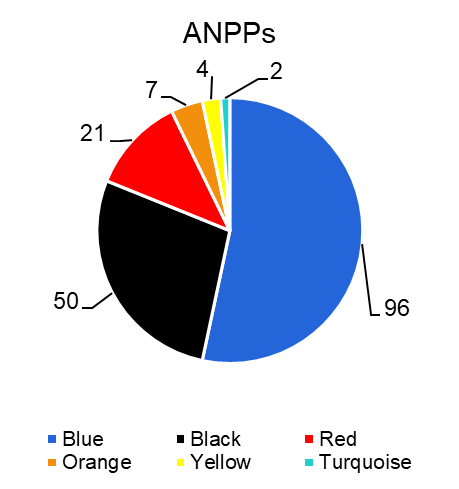


**Figure S1**. Colours of the MPs and ACPs identified in this work.

**Table S2**. Fitting results of size distribution data fitted to logistic functions (Eq. 3, fitting plot shown in the figure below) and mass concentrations calculated from size distribution.

|  | Fitting parameters  *size_med_* and *n* | Calculated mass concentration |
| --- | --- | --- |
| This work; single use PET bottles (in this work size = *d_v_*) | size*_med_* = 97.1 µm  (92.6 µm experimental median)  *n* = 3.51 ± 0.05 | 29-294 µm: 1.61 µg/L  < 100 µm: 143 ng/L  (169 ng/L experimental < 100 µm) |
| Oßmann et al. 2018, single se PET bottles | size*_med_* = 1.44 µm  *n* = 3.25 ± 0.51 | 23 ng/L  (0.4-10 µm) |
| Oßmann et al. 2018, reusable PET bottles | size*_med_* = 1.57 µm  *n* = 2.25 ± 0.36 | 171 ng/L  (0.4-10 µm) |
| Schymanski et al., 2018, single use bottles | size = 13.0 µm  *n* = 1.90 ± 0.49 | 260 ng/L  (5-100 µm) |
| Schymanski et al.,  2018, returnable bottles | size*_med_* = 9.8 µm  *n* = 2.07 ± 1.11 | 650 ng/L  (5-100) |

**
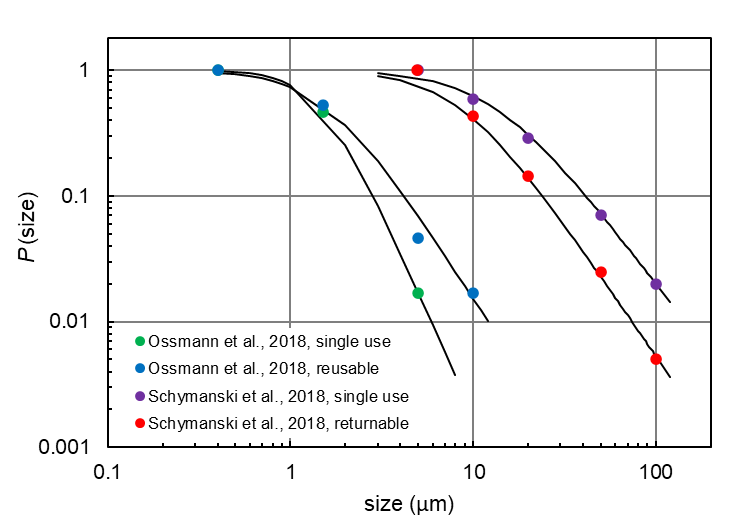
**

**Table S3**. Literature findings on MPs detected in bottled drinking water. Error in standard deviation (SD) or 95% CI, when disclosed.

| **Study details** | **Procedure** | **Results** | **Reference** |
| --- | --- | --- | --- |
| 21 brands of mineral water in PET and glass bottles from food stores in Germany. | EDTA added to dissolve calcium and magnesium particles; SDS to detach plastic particles from glass surface; filtration through 0.4 µm pore size aluminium coated polycarbonate membrane filters; analysis of subsamples using micro-Raman spectroscopy. | Single use PET: 2649 ± 2857 (SD) MPs/L; reusable PET 2689 ± 4371 (SD) MPs/L in new bottles and 8339 ± 7043 (SD) MPs/L in reused bottles; glass bottles 6292 ± 10521 (SD) MPs/L − 77.8-98.3 % of particles < 5 µm − PET dominant in PET bottles, and PE+PP in glass bottles. | Oßmann et al. 2018 |
| 22 returnable and single-use plastic bottles, 3 beverage cartons and 9 glass bottles from local stores in Germany. | Gold coated polycarbonate filter, 3.0 µm pore size; analysis using micro-Raman spectroscopy. | Single use: 14 ± 14 (SD) MPs/L; returnable: 118 ± 88 (SD) MPs/L; beverage cartons: 11 ± 8 (SD) MPs/L; glass bottles: 50 ± 52 MPs/L − ⁓80 % of MP particles had a particle size in the 5-20 μm range − 84 % of MPs in returnable plastic bottles were PES; PE was the most abundant polymer in glass bottles and beverage carton. | Schymanski et al. 2018 |
| 11 brands of bottled water in 11 different countries with different origin including filtered municipal water. | Identification with Nile Red followed by filtration using glass fibre 1.5 µm filters; ATR-FTIR used for some large particles (100 µm). | 93 % bottles with MP contamination − Average in the 3.7-2227 MPs/L range (6.5-100 µm) − PP was the most abundant polymer in particles > 100 µm − Fragments were the most abundant typology (66 %). | Mason et al. 2018 |
| 10 brands of still and sparking water in 0.5 L PET bottles (Italy). | 1 mL dispersed with 3 mL 65% HNO_3_ and 3 mL of water plus 9 mL dichloromethane. After centrifugation, extracts are dispersed with acetonitrile and visualized using SEM. | 100-3000 µg/L, median 657 ± 633(SD) µg/L (plastic density estimated from bottles) or 5.4 x 10^7^ MPs/L ± 1.95 x 10^7^(SD) MPs/L − Size in the 1.28-4.20 µm range (median 2.44 µm). | Zuccarello et al. 2019 |
| Single use PET bottles (10 brands, still and sparking) and glass bottles (3 brands, sparkling) from Thailand. | 0.45 μm and 20 μm cellulose filters; fluorescence microscopy (for particles < 50 μm), ATR-FTIR for > 50 μm, confocal Raman < 50 μm. | Average 81.0 ± 3.0 MPs/L (6.5–20 μm), 26.0 ± 2.0 MPs/L (20–50 μm) and 12.0 ± 1.0 MPs/L for ≥ 50 μm − Lower limit for fluorescence microscopy 6.5 μm − Fibres more frequent than fragments − Main polymers PET, PE, PP, and PA. | Kankanige and Babel 2020 |
| 11 brands in 0.5 L PET bottles sold in Iran. | Rose Bengal for organic particles followed by filtration 1.1 μm glass fibre; visual counting using stereomicroscope; ATR-FTIR and micro-Raman for selected particles. | 0-36 MPs/L, average 8.5 ± 10.2 MPs/L. | Makhdoumi et al. 2021 |
| 23 brands of PET-bottled water in China. | 0.4 μm gold-coated polycarbonate membrane filters and FPA micro-FTIR for polymer identification. | 2-23 MPs/bottle or 16 MPs/L − Most abundant MPs 100–300 µm (29–49 %) − Fibres represented form one third to 100 % − The proportions of MPs were PET (7 %), PE (6 %), PS (5 %), and PA (4 %) − Large number of cellulose fibres. | Zhou et al. 2021 |
| 16 brands of bottled water in Australia. | 5 μm polycarbonate filters and identification with Laser Direct Infrared (20-500 μm). | MPs in 94 % of the samples − Mean size 75 ± 22 μm − 3 ± 19 MPs/L ranging from 0 to 80 MPs/L − The most frequent polymer was PP (74 %) followed by PET, PA, PE and PS. | Samandra et al. 2022 |
| 8 single use bottled water brands in 1.5 PET bottles from Malaysia. | 0.45 μm filter paper; visual count and one filter paper per brand analysed using a compact FTIR spectrometer. | 8-22 MPs/L, average 11.7 ± 4.6 MPs/L, the highest corresponding to treated municipal water from processed river water − MPs < 1 mm accounted for 83%, MPs 100-300 μm 31 % − Polymers identified PET and PP. | Praveena et al. 2022 |
| 23 samples from 1.5 L PET bottles from Iran. | Wet peroxide oxidation 30% H_2_O_2_ + 0.05 M Fe(II) followed by 0.22 μm PTFE filtration, SEM for particle count and identification with micro-FTIR for mapping part of the filter surface. | 1497±1452 MPs/L (average),  200-6627 MPs/L (range) − 91.3 % MPs in the 1–10 μm range; 8.7 % in the 10–46.7 μm range − Polymers identified: PE, PET, PP, PVC, and PS. | Taheri et al. 2022 |
| 7 PET and 3 glass brands in China, domestic and foreign. | 10 μm stainless steel filters followed by LDIR analyses. | Average 72 ± 45 (SD) MPs/L, but cellulose was considered a MPs − Size 10-50 μm accounted for two-thirds, mostly films − Cellulose (68 %), PVC (17 %), and other polymers (15 %). | Li et al. 2023 |
| 50 brands commercially available in Turkey sold in PET and glass bottles. | 0.45 μm glass fibre filters, visual inspection using stereomicroscope and ATR-FTIR. | 2-35 MPs/L, average 4.6 ± 3.9 MPs/L (natural) and 12.6 ± 8.7 MPs/L (mineral) − Average size 105 ± 14 µm (range 8–316) in natural water and 64 ± 4 µm (range 12–184) in mineral water − PE and PP were the main polymers identified. | Altunışık 2023 |
| 13 brands of Spanish bottled water | 45 μm stainless-steel mesh filters, stereomicroscope inspection and micro-FTIR analysis. | Average 7.2 MPs/L − Size 45-5000 µm − Cellulose (86 %), PES, PU, PE and PP | Socas-Hernández et al., 2024 |
| 5 brands of still water representing ⁓40% market share in 1.5 PET bottles. | 0.8 μm black polycarbonate filters; identification using micro-FTIR. | 0.64-1.58 MPs/L, median 0.73 MPs/L − Median size 93 µm (76-130 µm, interquartile range) − PES, PE and non-natural cellulose were dominant. | This work |

***References for Tables S2 and S3.***

Aleksander-Kwaterczak U, Gaj D, Stelmach A, Wróbel TP. (2023). Investigating the content of microplastics and other extraneous particles in Polish bottled water. Geology, Geophysics and Environment, 2023, 49(4), 335–353.

Altunışık A. Microplastic pollution and human risk assessment in Turkish bottled natural and mineral waters. Environmental Science and Pollution Research 2023; 30: 39815-39825.

Kankanige D, Babel S. Smaller-sized micro-plastics (MPs) contamination in single-use PET-bottled water in Thailand. Science of The Total Environment 2020; 717: 137232.

Li H, Zhu L, Ma M, Wu H, An L, Yang Z. Occurrence of microplastics in commercially sold bottled water. Science of The Total Environment 2023; 867: 161553.

Makhdoumi P, Amin AA, Karimi H, Pirsaheb M, Kim H, Hossini H. Occurrence of microplastic particles in the most popular Iranian bottled mineral water brands and an assessment of human exposure. Journal of Water Process Engineering 2021; 39: 101708.

Mason SA, Welch VG, Neratko J. Synthetic polymer contamination in bottled water. Frontiers in Chemistry 2018; 6: 407.

Oßmann BE, Sarau G, Holtmannspötter H, Pischetsrieder M, Christiansen SH, Dicke W. Small-sized microplastics and pigmented particles in bottled mineral water. Water Research 2018; 141: 307-316.

Praveena SM, Shamsul Ariffin NI, Nafisyah AL. Microplastics in Malaysian bottled water brands: Occurrence and potential human exposure. Environmental Pollution 2022; 315: 120494.

Samandra S, Mescall OJ, Plaisted K, Symons B, Xie S, Ellis AV, Clarke BO. Assessing exposure of the Australian population to microplastics through bottled water consumption. Science of The Total Environment 2022; 837: 155329.

Schymanski D, Goldbeck C, Humpf H-U, Fürst P. Analysis of microplastics in water by micro-Raman spectroscopy: Release of plastic particles from different packaging into mineral water. Water Research 2018; 129: 154-162.

Socas-Hernández C, Miralles P, González-Sálamo J, Hernández-Borges J, Coscollà C. Assessment of anthropogenic particles content in commercial beverages, Food Chemistry 447, 139002, 2024.

Taheri S, Shoshtari-Yeganeh B, Pourzamani H, Ebrahimpour K. Investigating the pollution of bottled water by the microplastics (MPs): the effects of mechanical stress, sunlight exposure, and freezing on MPs release. Environmental Monitoring and Assessment 2023; 195: 62.

Zhou X-J, Wang J, Li H-Y, Zhang H-M, Hua J, Zhang DL. Microplastic pollution of bottled water in China. Journal of Water Process Engineering 2021; 40: 101884.

Zuccarello P, Ferrante M, Cristaldi A, Copat C, Grasso A, Sangregorio D, Fiore M, Oliveri-Conti G. Exposure to microplastics (<10 μm) associated to plastic bottles mineral water consumption: The first quantitative study. Water Research 2019; 157: 365-371.
